# Genetic counselors' professional identity in North America: A scoping review

**DOI:** 10.1002/jgc4.1931

**Published:** 2024-06-11

**Authors:** Kaye Stenberg, Rachel Mills, Isha Kalia, Lisa Schwartz

**Affiliations:** ^1^ Department of Biomedical Laboratory Sciences The George Washington University Ashburn Virginia USA; ^2^ MS Genetic Counseling Program University of North Carolina Greensboro Greensboro North Carolina USA; ^3^ The GW Faculty Medical Associates The George Washington University Washington DC USA

**Keywords:** diversity, equity and inclusion, genetic counselors, professional development, professional identity, scoping review, social identity theory

## Abstract

Professional identity (PI) comprises attributes, beliefs, values, motives, and experiences by which people define themselves in a professional role and evolves through socialization with others in the workplace. While there have been several studies exploring the expanding roles of genetic counselors, few have specifically addressed PI. This scoping review aimed to describe the contexts in which PI has been discussed or examined in the genetic counseling literature. Articles were searched using PubMed, Scopus, and CINAHL with a priori terms including and related to PI. Articles based in the United States or Canada and of all study designs, commentaries, and speeches were included. Date of publication was not restricted. Using social identity theory (SIT) to formulate a definition of PI, multiple reviewers applied inclusion and exclusion criteria to all titles, abstracts, and full‐text articles with conflicts addressed through consensus among all reviewers. A total of 5523 titles and/or abstracts were screened, and 467 full‐text articles were evaluated and categorized as (1) focusing on PI specifically, (2) containing elements of PI although focused on another topic, or (3) not related to PI. Eighty‐seven (87) articles were reviewed during the extraction phase. Ultimately, 41 articles were deemed to meet the agreed upon characteristics of PI. While empirical studies of PI among genetic counselors were limited, PI is being addressed in research focused on related areas, including professional development and diversity, equity, and inclusion, as well as in personal accounts, addresses, and commentaries. Sentiments regarding PI voiced by genetic counselors align with those reported among other health professionals. Given the lack of diversity in the field and rapidly expanding opportunities for genetic counselors, there is risk of some members of the profession feeling excluded, which in turn could negatively impact the collective identity of the profession and translate into impacts on patient care. Additional research regarding the PI of genetic counselors is needed.

**What is known about this topic**

- Professional identity (PI) comprises attributes, beliefs, values, motives, and experiences by which people define themselves in a professional role and evolves through socialization with others in the workplace.
- Genetic counselors have expressed feelings of exclusion from the profession, which could negatively impact the collective identity of the profession and patient care.

**What this paper adds to the topic**

- This is the first systematic review of PI within the genetic counseling literature.
- PI is addressed in genetic counseling literature in studies on topics tangential to PI or in personal accounts, addresses, and commentaries, but there is a paucity of empirical research specifically focused on PI.
- The existing literature is most often focused on areas related to PI, including a single person's perspective, professional development, diversity, equity, and inclusion, collective identity of the profession, reflective practice, ethics‐based, and historical accounts of the profession.

## 1 INTRODUCTION

Professional identity (PI) is defined as “the relatively stable and enduring constellation of attributes, beliefs, values, motives, and experiences in terms of which people define themselves in a professional role” (Schein, 1978, as cited in Ibarra, 1999, p. 764). PI evolves and adapts as professionals enter new roles through socialization with others in the workplace (Ibarra, 1999). A key component of PI is self‐categorization and a sense of belonging to a professional group (Tajfel & Turner, 2004). PI is in part derived by making comparisons, positive or negative, between oneself and others within the profession (ingroup) and outside of the profession (outgroup), respectively (Hogg & Terry, 2000).

The genetic counseling profession is at a turning point in its history, driven in large part by expanded opportunities for its members to work in areas in which they are not providing genetic counseling by its definition (Resta, 2006). In addition, according to the U.S. Bureau of Labor Statistics (2024), jobs within the genetic counseling profession are projected to grow by 16% in the current decade. Participants of several recent studies have noted a negative stereotype attributed to genetic counselors working in non‐direct patient care roles and/or have questioned whether or not they still consider themselves to be genetic counselors (Groepper et al., 2015; Schwartz et al., 2023; Strohmeyer et al., 2023; Zetzsche et al., 2014). If genetic counselors have a reduced sense of belonging within the profession or no longer consider themselves to be part of the ingroup of genetic counselors, it may result in a diffusion of identity of the profession overall, which in turn may negatively impact societal understanding and recognition of the profession (Fitzgerald, 2020; Hogg & Terry, 2000). A malalignment of PI, driven by a sense of exclusion from within the professional group, may result in reduced job satisfaction and increased stress among its members, and ultimately negatively impact patient care (Fitzgerald, 2020; Tajfel & Turner, 2004).

The literature pertaining to PI and its formation among other health professionals is vast and most often focuses on medical students (Chandran et al., 2019; Cruess et al., 2015; Sarraf‐Yazdi et al., 2021) and nurses (Cingel & Brouwer, 2021; Rasmussen et al., 2018). While there have been several studies exploring the expanding roles of genetic counselors in response to the growing availability of genomic testing and precision medicine (Christian et al., 2012; Cohen & Tucker, 2018; Field et al., 2016; McWalter et al., 2018; Waltman et al., 2016; Zetzsche et al., 2014), there is a paucity of empirical studies specifically addressing the issue of PI among genetic counselors.

Scoping and systematic reviews of PI in other specific health professions have been conducted (Cornett et al., 2023; Matthews et al., 2019; Schubert et al., 2023), but a systematic review of PI within the genetic counseling literature had not been performed previously. In a scoping review of PI research in health professions in general, Cornett et al. (2023) found 160 studies across 17 health professions, but genetic counseling was not among them. To inform future research of PI among health professionals, Cornett et al. (2023) identified multiple constructs among the resultant papers, which they organized into five major themes: the lived experience of PI, the world around me, belonging, me, and learning and qualifications. Over half of the studies (52%) were focused on the construct of “becoming from performing” within the lived experience of PI theme. These studies focused primarily on professional identity formation (PIF) of trainees and early career practitioners as they develop a sense of being a health professional through observation and role modeling of more experienced professionals, while they receive and integrate feedback through a reflective process (Cornett et al., 2023).

This article details the process and findings of a scoping review that was conducted to (1) describe the contexts in which concepts related to PI have been discussed or examined in the genetic counselor profession, (2) identify research gaps in the existing literature, and (3) inform the development of an interview protocol for a qualitative study exploring PI among genetic counselors. Unlike a systematic review that aims to critically examine evidence to inform practice, a scoping review intends to identify how key characteristics related to a concept (e.g., factors related to perceptions of PI; Rasmussen et al., 2018) are being described, defined, and studied in existing literature (Munn et al., 2018).

It was anticipated that there would be limited existing literature focused specifically on the PI of genetic counselors; therefore, the scoping review used a priori search terms related to professional identity, including professional development, scope of practice, career satisfaction, and meaning making, as well as post hoc terms identified during the review, to fully map existent literature and identify gaps related to other constructs of PI. In addition, the scoping review was guided by social identity theory (SIT) as a framework to explore how PI has been described among genetic counselors (Harwood, 2020; Tajfel & Turner, 2004). SIT has been used as a lens through which to study PI among nurses (Willetts & Clarke, 2014) and other health professionals as identified by Cornett et al. (2023). A key construct of SIT is a sense of belongingness to a group (Tajfel & Turner, 2004), and thus, we used the following characteristics of PI to guide review of full‐text articles: one's connectedness, sense of belonging/exclusion from the group, confidence in one's abilities, socialization with other genetic counselors, distinction from other specialists/providers, and reason for joining/leaving the profession. This construct did not include what the study team considered to be the identity of the *profession* of genetic counseling (collective professional identity): shared training, values and ethics, knowledge and skills, competencies, professional organizations, licensure, and certification.

## 2 METHODS

Using the PRISMA Extension for Scoping Reviews (PRISMA‐ScR) Checklist (Tricco et al., 2018), articles related to PI of genetic counselors were searched. In January 2023, MedLine, Scopus, and CINAHL databases were searched for records of peer‐reviewed articles using the following phrases: (sense of belonging OR social identification OR nontraditional OR non‐traditional OR transition OR professional OR career OR occupation OR vocation OR calling OR meaning OR confidence OR self‐identification OR identit* OR self‐identification) AND (genetic counsel*). Articles were restricted to those based in the United States and Canada. There were no restrictions imposed on study design, type, or publication date. Results from each database were imported into the Covidence web application for review. Duplicates not identified by Covidence were removed manually.

Two out of three reviewers (JS, RM, and LS) independently identified those titles and/or abstracts that met the following eligibility criteria: related to setting/roles, training, supervision and training of supervisors, or professional development, speeches and commentaries, introduction to special editions of the Journal of Genetic Counseling, ethics, and history of the profession. Titles and abstracts for which the full‐text article could not be retrieved or were full books or chapters within books were excluded. Consensus was reached through discussion among three reviewers (KS, JM, and LS).

During the next phase, relevant full‐text articles were screened by two out of four reviewers, with consensus made by all four reviewers (KS, JM, IK, and LS). At this phase, articles were labeled as being core if PI was the purpose of the article or peripheral if constructs of PI emerged in the article, but PI was not the explicit focus of the article. In addition, articles were further categorized as being (1) single person's perspective; (2) diversity, equity, inclusion, and justice (DEIJ); (3) professional development; (4) identity of the profession (collective); (5) ethics; (6) training and continuing education; (7) historical; (8) reflective practice; and/or (9) supervision. Full‐text articles that focused on professionalism or clinical practice/provision of genetic counseling services were excluded. While they are closely related, professionalism has been distinguished from PI, with professionalism being the outward conduct of professional behaviors as witnessed by others (e.g., patients, society), and PI is the internalizing of a professions' values and norms that occurs on the individual level that results in one “thinking, acting, [and] feeling like a professional” (Moseley et al., 2021, p. 13). The scoping review also aimed to focus on genetic counselors' perspectives regarding their connection to the profession and not their roles as genetic counselors.

During the extraction phase, articles were assessed for eligibility and full‐text articles were included in the final compilation of articles if they met the following characteristics of PI: one's connectedness, sense of belonging/exclusion from the group, confidence in one's abilities, socialization with other genetic counselors, distinction from other specialists/providers, and reason for joining/leaving the profession. Using an extraction table built within Covidence, all full‐text articles were identified as being core or peripheral and were labeled by study design, empirical (quantitative, qualitative, or mixed methods) or non‐empirical. Furthermore, each article was tagged as being a defining moment series article, NSGC awardee address, NSGC Presidential Address, commentary/opinion piece/letter to the editor, DEIJ, reflective practice, professional development, ethics‐based, and/or historical. Articles could have more than one tag. For each article, the context in which PI was described and illustrative quotes were extracted.

## 3 RESULTS

**FIGURE 1:**
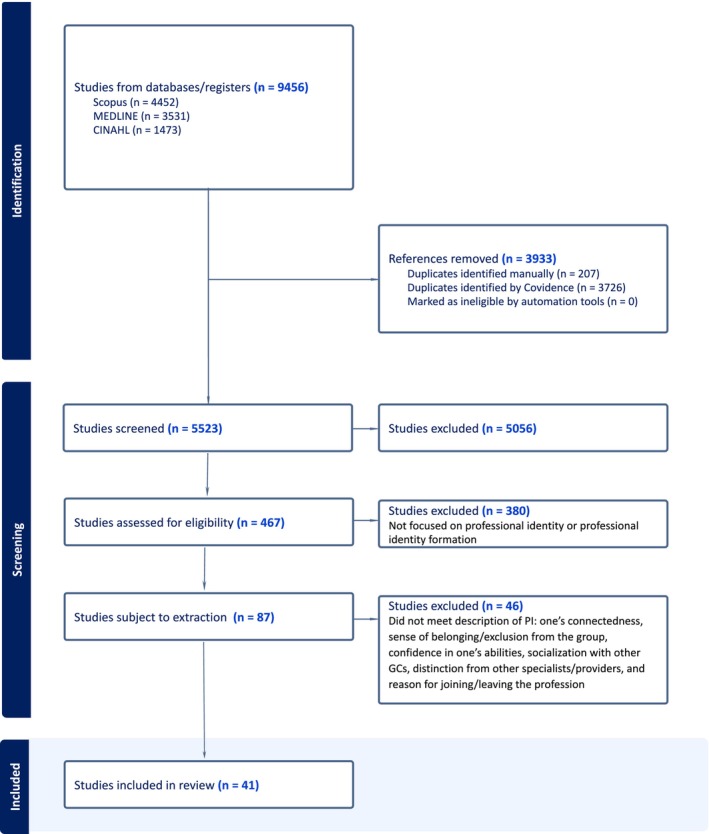
PRISMA‐ScR diagram. All excluded articles were assessed by at least two independent reviewers.

The initial search yielded 9456 articles, but after removing duplicates, 5523 titles and abstracts were reviewed using the defined inclusion and exclusion criteria. This step resulted in an additional 5056 titles and abstracts being excluded, leaving 467 articles for full‐text review. Four hundred and sixty‐seven (467) full‐text articles were assessed for eligibility, and 380 studies were excluded because they either did not focus on PI or PI formation, or did not meet other inclusion criteria. Eighty‐seven (87) articles were subject to further review during the extraction phase (see Figure 1).

Ultimately, 41 articles were deemed to meet the study team's agreed upon characteristics of PI. Among the final 41 articles, each article was labeled with tags based on its relevance (core or peripheral), study design, and category/tag. Table 1 provides a summary of the characteristics of the 41 full‐text articles, which are further described below. Table S1 provides additional information about each article.

**TABLE 1:** Full‐text article characteristics.

*N* = 41	*n*	%
Relevance		
Peripheral	32	78
Core	9	22
Study design		
Non‐empirical	22	54
Empirical	19	46
Qualitative	14	74
Quantitative	3	16
Mixed methods	2	10
Categories^a^		
(1) Single person's perspective	17	41
Defining moments	11	65
NSGC presidential address	4	24
NSGC awardee address	1	6
Commentary/opinion piece/letter to editors	1	6
(2) Professional development	16	39
(3) Diversity, equity, inclusion, and justice (DEIJ)	10	24
(4) Collective identity of the profession	9	22
(5) Reflective practice	6	15
(6) Ethics‐based	2	5
(7) Historical	1	2

^a^ Articles could be labeled as more than one category, so equals more than 100%.

### 3.1 Article relevance

Nine of the 41 articles (22%) were considered to be core, as the primary focus of the article was PI (Baty, 2018; Bradley, 2012; Carmichael et al., 2021; Finucane, 2012; Hippman & Davis, 2016; Miranda et al., 2016; Ramos, 2018; Strohmeyer et al., 2023; Uhlmann, 2012). Of the remaining 32 (78%) articles, PI was described but was not the primary focus of the article and thus were labeled as peripheral (Abrams & Kessler, 2002; Atzinger et al., 2007; Bao et al., 2020; Bartlett & Johnson, 2009; Cantor et al., 2019; Channaoui et al., 2020; Curnow, 2012; Darr et al., 2023; Davis et al., 2020; Disco, 2016; Flynn, 2012; Geller et al., 2009; Groepper et al., 2015; Infante, 2012; Knutzen, 2012; Lakhani, 2012; Liker et al., 2019; Liu et al., 2011; Matloff, 2006; McKanna, 2012; Means et al., 2020; Oswald, 2012; Paull & Lipinski, 2012; Peters, 1987; Ramachandra et al., 2019; Riordan, 2021; Runyon et al., 2010; Schoonveld et al., 2007; Sturm, 2019; Wells et al., 2016; Zahm et al., 2016; Zetzsche et al., 2014).

### 3.2 Article study design

The majority (*n* = 22; 54%) of the 41 articles were non‐empirical (Abrams & Kessler, 2002; Bao et al., 2020; Baty, 2018; Bradley, 2012; Channaoui et al., 2020; Curnow, 2012; Disco, 2016; Finucane, 2012; Flynn, 2012; Infante, 2012; Knutzen, 2012; Lakhani, 2012; Liu et al., 2011; Matloff, 2006; McKanna, 2012; Oswald, 2012; Paull & Lipinski, 2012; Peters, 1987; Ramos, 2018; Riordan, 2021; Sturm, 2019; Uhlmann, 2012). Among the 19 articles that used an empirical study design, 14 (74%) were qualitative (Atzinger et al., 2007; Bartlett & Johnson, 2009; Carmichael et al., 2021; Darr et al., 2023; Davis et al., 2020; Hippman & Davis, 2016; Liker et al., 2019; Means et al., 2020; Miranda et al., 2016; Ramachandra et al., 2019; Schoonveld et al., 2007; Wells et al., 2016; Zahm et al., 2016; Zetzsche et al., 2014), three (16%) were quantitative (Groepper et al., 2015; Runyon et al., 2010; Strohmeyer et al., 2023), and two (10%) used mixed methods (Cantor et al., 2019; Geller et al., 2009).

### 3.3 Article categories

All 41 articles were tagged to divide them into categories. Some articles had multiple tags and thus were included in more than one category.

#### 3.3.1 Single Person's perspective

The largest category was single person's perspective (17; 41%), consisting of 11 (65%) articles from the *Journal of Genetic Counseling* (JOGC) defining moment series (Bradley, 2012; Curnow, 2012; Flynn, 2012; Infante, 2012; Knutzen, 2012; Lakhani, 2012; McKanna, 2012; Oswald, 2012; Paull & Lipinski, 2012). In their introduction to the series, Veach and LeRoy (2012) noted that the compiled articles were in response to a call for authors “to describe the nature of a defining moment and how it affected their professional identity and practice (p. 277).” Each article described experiences, both personal and professional, which impacted the author's sense of themselves as a genetic counselor. For example, Bradley (2012) shared her failure of the ABGC examination, and subsequent PI crisis, as being a defining moment in her career as a genetic counselor:

Failing the test called all of my early successes and development into question, and I found myself facing a professional identity crisis (p. 175)… It wasn't an easy lesson, but in the last year I learned that certification does not define me as a genetic counselor, I define myself (p. 376).

While not specifically part of the JOGC defining moments series, Matloff (2006) and Disco (2016) also shared key defining moments in their personal lives that impacted their professional lives and thus PI. Disco (2016) shared her personal story as to why she chose being a genetic counselor as a career path as well as the profession's overlap with her previous freelance career in theater lighting. She stated “I have come to believe that ways of thinking about work from my years in theater lighting are also fundamental to genetic counseling (p. 683).”

Four of the 17 (24%) articles tagged as single person's perspective were NSGC presidential addresses (Finucane, 2012; Ramos, 2018; Riordan, 2021; Sturm, 2019) and one (6%) was an NSGC awardee address (Uhlmann, 2012). In these articles, the authors often shared their own career trajectories as genetic counselors and how their experiences shaped their PI. In her NSGC Presidential Address, Riordan (2021) described elements of her personal identity that influenced her work and how her work as a genetic counselor had shaped her. She shared what motivated her to pursue being a genetic counselor as a profession, “It was then, when I discovered that a profession existed that so masterfully blended the study of science and the art of communication, that I had the crystal‐clear revelation. I wanted to be a genetic counselor (p. 110).”

In her NSGC Presidential Address, Finucane (2012) noted that despite serving in what many may have considered at the time to be an atypical role and setting she still maintained her identity as a genetic counselor. She had been working in primarily educational settings with nonmedical colleagues, but she utilized core skills of genetic counselors, including in‐depth knowledge of genetics and healthcare delivery, strong interpersonal skills, communication, research skills, and self‐awareness:

As you can see, my path has taken me in a direction that, prior to my earlier presidential decree, one might have called “nontraditional”, and yet I've always identified myself as a genetic counselor. I may have pondered the rhetorical question [Am I still a genetic counselor?], but really, I know I'm a genetic counselor and I've always been one (p. 5).

Finally, as the one (6%) commentary/opinion piece/letter to the editors to the JOGC among the 17 articles in this category, Peters (1987) described principles of genetic counseling that align with feminist theory and in turn the identity of the profession.

#### 3.3.2 Professional development

Sixteen (16; 39%) of the 41 articles focused on professional development of genetic counselors (Atzinger et al., 2007; Curnow, 2012; Davis et al., 2020; Disco, 2016; Hippman & Davis, 2016; Lakhani, 2012; Miranda et al., 2016; Oswald, 2012; Paull & Lipinski, 2012; Ramachandra et al., 2019; Riordan, 2021; Runyon et al., 2010; Strohmeyer et al., 2023; Wells et al., 2016; Zahm et al., 2016; Zetzsche et al., 2014). Zahm et al. (2016) conducted a qualitative study involving 34 genetic counselors of varying levels of experience after completing their master's degree training: novice (0–5 years), experienced (6–14 years), and seasoned (>15 years). Three main themes were identified through data analysis, all of which were framed in context with their evolving perception as being a clinician: (1) relationship with clinical work, (2) relationship to PI, and (3) relationship to development of the profession. The domains within the PI theme revolved primarily around current motivations to practice genetic counseling, definitions of professional development, and career satisfaction.

The mixed‐methods study conducted by Runyon et al. (2010) focused on what genetic counselors learn about themselves while working as a genetic counselor. In interviews of select survey respondents, one participant shared:

Genetic counseling has helped me shape the definition of myself. I've learned how I feel ethically on issues, how I react/cope, how I make decisions, and how I grieve. I've learned that I treasure family, friends, and community. Much of my identity is intertwined with my profession (p. 376).

Hippman and Davis (2016) conducted qualitative interviews exploring the experiences of genetic counselors who made career transitions, and in particular, how that change impacted their sense of PI and inclusion among other genetic counselors. As one participant noted:

I felt very judged for going into research immediately following graduation, like I was letting the team down or like my spot in the training program had been wasted because I wasn't going to be a part of the clinical genetic counselor workforce. To be clear, I judged myself, but I also felt judged by other genetic counselors (p. 727).

Strohmeyer et al. (2023) used a survey to assess characteristics of laboratory and industry roles and perceptions among genetic counselors working in these settings. They found that genetic counselors in laboratory and industry roles were experiencing PI issues, stigmatization, and overall not feeling like a genetic counselor because of their job position.

In a qualitative study, Atzinger et al. (2007) investigated the opinions and attitudes of genetic counselors with doctoral degrees and how it has impacted their careers. While obtaining a doctoral degree did not significantly change the practice of most participants, genetic counselors with a doctoral degree reported feeling that they received more respect from colleagues. A participant shared, “One thing that frustrates genetic counselors that I have worked with somewhat is sometimes feeling a little bit of a lack of respect, and I don't really sense that as much” (Atzinger et al., 2007, p. 232). In a similar article focused on the career trajectories of genetic counselors with advanced skills, Davis et al. (2020) found that the factors that influence professional development also influence PI. As noted in the article:

Several interviewees highlighted how deliberate decision‐making was facilitated by the appeal of an expanded professional identity. They imagined themselves as professionals with greater capabilities, garnering greater respect, accomplishing more; these aspirational identities intertwined with evolving motivations and goals to which they purposefully matched advanced training that would help them achieve those identities and described how others could do so (p. 778).

#### 3.3.3 Diversity, equity, inclusion, and justice (DEIJ)

Ten (10; 24%) of the 41 articles focused on issues related to diversity, equity, inclusion, and justice (DEIJ; Bao et al., 2020; Bartlett & Johnson, 2009; Cantor et al., 2019; Carmichael et al., 2021; Channaoui et al., 2020; Darr et al., 2023; Geller et al., 2009; Liu et al., 2011; Peters, 1987; Schoonveld et al., 2007). Using focus groups, Carmichael et al. (2021) characterized the training experiences of genetic counseling students who identified as racial or ethnic minorities and how those experiences impacted participants' sense of belonging in the genetic counseling profession and at the NSGC conference. Participants described “interactions between participants and their classmates, faculty, clinicians, or other members of the profession that negatively impacted their sense of belonging in the profession, causing them to feel ‘othered’ (p. 822).” Similarly, in Bao et al. (2020), four genetic counselors shared individual reflections on how personal cultural identity and the lack of diversity within the profession affects its members. One author recognized that she benefited from her personal identity as a White woman of middle/upper‐middle socioeconomic class given the profession's messaging that its members must conform to White behaviors and beliefs:

While there is, of course, a need for adherence to a common set of practice standards as with any licensed health care profession, I continue to be surprised at how much this seems to spill over into personal attributes in our field as if we all need to be similar in personality in order to collectively gain a foothold as a profession (p. 318).

Channaoui et al. (2020) presented the creation of the NSGC Diversity and Inclusion Task Force and its recommended charges for creating a more diverse, inclusive, and equitable genetic counseling field. They noted, “There is a need to promote and leverage a culture of inclusivity that supports visible and invisible diversity to expand perspectives represented in the field. (p. 193).” In a mixed‐methods study focused in the impact of religious beliefs and values among genetic service providers, including genetic counselors, Geller et al. (2009) found that “the need to hide their beliefs from their colleagues is a source of distress for religiously oriented genetics professionals and may undermine their sense of belonging and commitment to the profession (p. 38).” Schoonveld et al. (2007) explored the factors that may serve as barriers or facilitators to entering the profession among genetic counselors who were underrepresented in the field, including due to gender:

All three male students and some of the practicing counselors expressed anxiety about how they actually fit into the field… Since the males had spent most of their lives in the majority vis a vis their gender, their more recent minority status as graduate students might be more distressing than for the female student participants who have a history of being in the minority because of their ethnicity (p. 63).

Cantor et al. (2019) studied the training experiences of genetic counseling students and recent graduates with mental illness. They found that participants “described trying to live up to an ideal vision of a genetic counselor, and how the self‐doubt that this created interfaced with mental illness (p. 351).”

#### 3.3.4 Collective identity of the profession

Nine (9; 22%) of the 41 articles were characterized as being focused on the collective identity of the profession (Baty, 2018; Finucane, 2012; Knutzen, 2012; Means et al., 2020; Peters, 1987; Ramos, 2018; Riordan, 2021; Sturm, 2019; Uhlmann, 2012). These articles, although containing elements of individual PI, also described features that the study team considered to be belonging to the profession as a whole, such as a historical account of key milestones in the development of the genetic counseling profession (Baty, 2018), expanding roles for genetic counselors (Finucane, 2012), and board certification (Knutzen, 2012).

#### 3.3.5 Reflective practice

Six (6; 15%) of the 41 articles were tagged as focusing on reflective practice (Abrams & Kessler, 2002; Liker et al., 2019; Matloff, 2006; McKanna, 2012; Wells et al., 2016; Zahm et al., 2016). Using vignettes, Abrams and Kessler (2002) noted “each genetic counselor can tell his or her own stories, his or her own struggle to find a place for oneself, to fulfill some personal wish or hope, to find ways of working effectively within a complex system with other human beings with their own ego needs, inner struggles, and self‐doubts (p. 16).” Matloff (2006) explained how her mother's diagnosis with breast cancer impacted her practice and perspective. “I've learned that my personal experiences will shape my counseling style, my career, and my interactions with patients and colleagues. I've also learned that this is okay (p. 143).”

#### 3.3.6 Ethics‐based

Two (2; 5%) of the 41 articles were tagged as being ethics‐based (Groepper et al., 2015; Liker et al., 2019). In the quantitative study by Groepper et al. (2015) examining genetic counselors working in laboratory settings, PI issues were among the ethical/professional domain choices in their survey, which was then further delineated into categories, one of which was professional image. In an open‐ended response, one participant described “the pervasive negative attitude and misconceptions that academic genetic counselors have towards laboratory‐based genetic counselors, an ‘us vs. them’ mentality, which can impact patient care (p. 587).”

#### 3.3.7 Historical

Finally, one of the 41 articles (2%) by Baty (2018) provided a summary of the development of the genetic counselor as a distinct profession. Shared milestones, including the establishment of a graduate program, a national society, certification examination, a code of ethics, and research and scientific publications, “contributed to the identity of the genetic counselor as a uniquely qualified professional (p. 54).” However, she went further to note that “the diversification of roles and career paths raises the issue of genetic counselor identity. In earlier days of the profession, the term genetic counselor referred to a set of roles regardless of education or professional identity (p. 59).”

## 4 DISCUSSION

This scoping review revealed that genetic counselors' PI has been addressed in the literature, mainly through single person perspectives or focused on issues tangential to PI such as professional development or DEIJ. However, there have been very few empirical studies that have specifically addressed genetic counselors' PI (Carmichael et al., 2021; Hippman & Davis, 2016; Miranda et al., 2016).

As noted earlier, a primary aim of the scoping review was to inform the development of an interview protocol for a qualitative study exploring PI among genetic counselors. One challenge to conducting further empirical research on the topic of PI among genetic counselors is that PI is complex and often not well defined. Fitzgerald (2020) performed a concept analysis, similar to a scoping review, of the health‐related professional literature in which PI was empirically studied and explicitly defined. Among 68 articles included in her analysis, she found the following common themes: (1) shared characteristics, including actions and behaviors, knowledge and skills, and values, beliefs, and ethics; (2) context and socialization; and (3) group and personal identity. These themes are similar to those described in the scoping review by Cornett et al. (2023) noted earlier. As detailed below, the resultant articles from our scoping review align with the themes identified by Fitzgerald (2020) and Cornett et al. (2023), and can guide future research regarding PI among genetic counselors.

For example, many articles fit into Fitzgerald's (2020) theme of shared characteristics, as well as Cornett et al.'s (2023) theme of the belonging, which were encapsulated by descriptions of common traits or experiences that may be shared by many genetic counselors or the profession as a whole (Bao et al., 2020; Baty, 2018; Bradley, 2012; Curnow, 2012; Disco, 2016; Flynn, 2012; Infante, 2012; Knutzen, 2012; Liu et al., 2011; Means et al., 2020; Miranda et al., 2016; Oswald, 2012; Paull & Lipinski, 2012; Peters, 1987; Ramos, 2018; Riordan, 2021; Runyon et al., 2010; Wells et al., 2016). As noted by Ramos (2018) in her NSGC Presidential Address:

We share the same passions: A passion for science, for helping people, for improving human health, and let's be honest for cardigans. That is why “I am a genetic counselor” is so powerful. It unites us. In our common background, our passions, our values, and our shared vision (p. 2).

Yet, shared characteristics among genetic counselors were not always viewed positively and could result in a reduced sense of belonging among some members within the profession. In Bao et al. (2020) one author noted, “Not only do most of us share a similar skin color, but there seems to be a tendency to dress, think, speak, and act similarly (p. 318).” While acknowledging that having shared values is important to the profession, a participant in one of the few empirical studies specifically focused on PI among genetic counselors (Carmichael et al., 2021) noted:

We want to make sure that we create this community moving forward to be more inclusive, to be non‐exclusionary and be intentional about that. Because of the fact of a lot of these perceived identities to be a genetic counselor, it becomes a barrier for us to get more people of diverse backgrounds in our field… There are certain values that we can be homogenous in, but not necessarily in personality (p. 820).

Therefore, future research regarding genetic counselors' PI, as related to their sense of belonging, must include the delineation of the key characteristics, including actions and behaviors, knowledge and skills, and values, beliefs, and ethics, that genetic counselors hold in common despite differences in personal traits, professional roles, and practice settings.

Aligned with Fitzgerald's (2020) theme of context and socialization and Cornett et al.'s (2023) theme of the lived experience, three articles focused on one's engagement with colleagues (Liker et al., 2019; Sturm, 2019; Uhlmann, 2012). Liker et al. (2019) conducted interviews with genetic counselors who offer infertility counseling. Participants described how their experiences shaped their professional and personal decisions as a genetic counselor as well as their practice and attitudes. They felt well supported in their roles by genetic counselor and non‐genetic counselor colleagues.

In their addresses to the NSGC membership, both Sturm (2019) and Uhlmann (2012) noted how their connections with fellow genetic counselor colleagues were instrumental in their own careers. Sturm (2019, p. 5) noted, “These types of bonds allow us, as genetic counselors, to support each other and cheer each other on, through close‐knit, collegial relationships, and often deep friendships, via our shared experiences and challenges. Sister chromatids—you've empowered me.” Thus, the manner and degree to which genetic counselors view their colleagues, both fellow genetic counselors and others, and professional organizations such as the NSGC as supportive of their PI should be explored in future research.

The theme of the lived experience, highlighted by Cornett et al. (2023), also emphasized the importance of the genetic counselor and patient interaction contributing to PI development. This concept is supported by a defining moment from Flynn (2012, p. 186) where she recounted, “Serving as this couple's genetic counselor allowed me to experience a ‘Defining Moment’ in my career by working with families during their most trying times and functioning as a support, I fulfill my role as a genetic counselor while developing into a better one.”

Articles discussing PI in our scoping review were reflective of Fitzgerald's (2020) theme of personal and group identity, as well as Cornett et al.'s (2023) themes of me and the world around me. Genetic counselors described a conflict between their personal identity and what they perceived as the group identity of the profession (Abrams & Kessler, 2002; Bao et al., 2020; Bartlett & Johnson, 2009; Cantor et al., 2019; Carmichael et al., 2021; Channaoui et al., 2020; Darr et al., 2023; Geller et al., 2009; Lakhani, 2012; Matloff, 2006; McKanna, 2012; Schoonveld et al., 2007). For example, in a study focused on genetic counselors with disabilities, Darr et al. (2023) found some participants did not feel that their personal identity aligned with that of the group identity of the profession:

This idea of what kind of person a genetic counselor is that's often said in a jokey way, like, ‘Oh, genetic counselors are Type A women in cardigans’ and it's like a stereotype or a joke, but there were definitely times where I felt like, ‘Do I even belong in this profession’ in terms of the kind of person I am, how my brain works, I kinda felt like sometimes I don't know if this is the right fit for me, I don't know how I'm going to function in this world. But ultimately, I felt like those aren't big enough barriers to keep me from doing what I wanted in the field (p. 243).

Aligned with Cornett et al.'s (2023) theme of learning and qualifications, Atzinger et al. (2007) reported that “many of the genetic counselors who hold a PhD that were interviewed believe the doctoral degree in genetic counseling is of potential benefit by providing the individual with a wider base of knowledge in research or another specialty area and by fostering greater respect and more opportunities” (p. 237). Yet, Davis et al. (2020) reported:

Some interviewees noted that concerns regarding a loss of identity as a GC [genetic counselor] acted as a barrier to decision‐making about making a change, particularly in cases where additional training could draw someone away from core genetic counseling tasks such as clinical care or alter one's sense of self to that of another professional. Regret about loss of GC identity varied among GCs, as well as within individual GCs over the course of their careers (p. 779).

A significant number of articles also addressed the impact of the expanding roles for genetic counselors resulting in a disconnect between one's individual PI and the group identity of the genetic counselor profession (Atzinger et al., 2007; Davis et al., 2020; Finucane, 2012; Groepper et al., 2015; Hippman & Davis, 2016; Ramachandra et al., 2019; Strohmeyer et al., 2023; Zahm et al., 2016; Zetzsche et al., 2014). In a study of genetic counselors entering the workforce, Ramachandra et al. (2019) identified a recurrent theme among all participants of a lack of confidence and imposter syndrome, but for those in non‐patient facing roles, doubt in one's clinical skills was more acute. This finding was not limited to novices to the field, as Davis et al. (2020) found that choices regarding one's career path also influenced genetic counselors' professional identity and sense of belongingness to the profession.

The impact of expanding roles on PI among other health professionals has been documented (Piil et al., 2012; Thompson et al., 2018). Thompson et al. (2018) conducted a qualitative study of nurses who were working in nursing homes. They described this setting as different from traditional roles since nursing home nurses attended to the social well‐being of their patients rather than only their healthcare needs, which negatively impacted their PI. Similar to findings described within the genetic counseling literature (Groepper et al., 2015; Strohmeyer et al., 2023; Zetzsche et al., 2014), nursing home nurses expressed feelings of being isolated and excluded—‘us versus them’—from the remainder of the nursing profession (Thompson et al., 2018). Yet, shared characteristics, like training, actions, and values, allowed participants to maintain a sense of professional identity as a nurse: “And I think we're all doing the same training—because we're working, we're doing the same thing. We should be you know, we all have the same goals and the same standards and things like that” (p. 1059).

Piil et al. (2012) explored the impact of expanding roles among nurses working in areas that were traditionally reserved for physicians. Similar to Davis et al. (2020), nurses in this study expressed “a higher sense of autonomy, self‐esteem, and confidence in their practice… [which had] a positive impact on their professional identity” (p. 329).

As noted earlier, Baty (2018) provided a historical account of the growth of the genetic counselor profession. She acknowledged that expansion of practitioners' roles and practice areas offers many advantages, such as increased salary, responsibilities, and application of professional skills, but that “functioning in roles outside the typical genetic counselor identity” (p. 56) is among several *disadvantages* (emphasis added). Baty went on to question whether genetic counselors who attain advanced skills that are not typically associated with genetic counseling, such as leadership, management, or research, still identify as genetic counselors or instead identify with other professionals in similar roles. She offered anecdotal evidence that genetic counselors with roles different from typical genetic counseling roles still identify as genetic counselors and concluded that “with more diverse career paths utilizing genetic counseling core and advanced skills, we may see more diversity of genetic counselor identity (p. 59).” However, a ‘typical’ genetic counselor identity has not been reported nor has the possible diversity of genetic counselors' professional identities been explored. In fact, a participant in the Davis et al. (2020) study of genetic counselors with advanced skills reflected:

I think many of us who've been in industry for a long time or who have left clinical jobs to go to industry… you've gone to the dark side. There's this feeling ‘you're not doing what you're trained to be doing.’ And I think that's a natural feeling, but for NSGC to be inclusive of all genetic counselors regardless of their role, would go a long way to people like me and others who have forged new paths and still identify as a genetic counselor (p. 779).

Thus, it is critical to further examine the professional identity of genetic counselors as the profession experiences expansion in terms of the number and diversity of its members and emergence of roles not previously held by genetic counselors.

### 4.1 Limitations

This scoping review only focused on the peer‐reviewed literature in journal articles, and therefore, relevant work from books, unpublished reports, and gray literature may be missing. The search was limited to articles published by authors in the United States and Canada to ensure commonality in training and practice, despite differences in healthcare systems, yet the issue of PI among genetic counselors has been discussed outside of these geographical areas. For example, McEwen and Jacobs (2021) described implementing the development of a philosophy of practice statement into their master's in genetic counseling program in Australia. They noted the value of developing one's philosophy of practice statement, particularly as opportunities for genetic counselors to work in diverse settings increase:

As genetic counselors embrace the opportunities of increasingly diverse positions in healthcare systems, it is crucial that they are confident in their professional identity. We believe that through reading, discussing, writing and reflecting on what we do and who we are as we develop and modify our personal philosophies of genetic counseling, we deepen our understanding of ourselves and our roles as individual genetic counselors and our identity as a profession (p. 9).

Thus, there may be a number of papers addressing genetic counselor PI in other regions of the world in which the genetic counselor profession is emerging that were not included in our scoping review.

Finally, PI is a complex construct with numerous definitions in the literature. Although attempts were made to be systematic in our review, including use of all study team members to create consensus, each member of the research team has varying degrees of training, experience, and expertise in genetic counseling and the professional identity literature, which may have impacted how they applied the inclusion and exclusion criteria.

### 4.2 Implications for research and practice

This scoping review demonstrates that there is a paucity of empirical research focused on genetic counselors' PI. Among the articles identified, genetic counselors have expressed concerns regarding their PI, including a decreased sense of belonging in the profession due to conflicts in personal identity and the impact of expanding roles. These sentiments may lead to decreased career satisfaction, attrition from the field, and diffusion of identity within and outside of the profession, all of which may negatively impact patient care.

This scoping review also revealed a lack of literature addressing professional identity formation (PIF) among genetic counselors, which was identified as one of the major contributing factors of PI across multiple health professions (Cornett et al., 2023). Other health professions have conducted research related to PIF (Lewis et al., 2023; Moseley et al., 2021), but research on the formation of genetic counselor PI is limited (Mills, 2024). Furthermore, empirical research on the PIF of genetic counselors, including the impact of supervision, training, and continued education, is essential for a better understanding and support of PIF within the genetic counseling profession. In addition, as the genetic counseling profession continues to develop and expand in countries outside of North America, replicating the scoping review in these regions may further our understanding of the PI of genetic counselors globally.

Future research focused on the PI of genetic counselors is currently underway by our study team with funding from the NSGC Jane Engelberg Memorial Fellowship (JEMF). The findings of this scoping review are being incorporated into the second phase of the JEMF‐funded study, which involves thematic analysis of 50 qualitative interviews with genetic counselors from diverse backgrounds in various practice settings and roles. The scoping review guided the development of our interview protocol by highlighting Fitzgerald's (2020) themes, such as the potential impact of shared characteristics, context and socialization, group, and personal identity. In addition, the constructs of PI identified in the health professions literature by Cornett et al. (2023), such as belonging and the lived experience, will inform deductive coding and thematic analysis.

## 5 CONCLUSION

Our scoping review of the genetic counseling literature found that while empirical studies of PI among genetic counselors are limited, the topic is being addressed in research focused on related areas, including professional development and DEIJ, as well as in personal accounts, NSGC addresses, and commentaries. Sentiments regarding professional identity voiced among genetic counselors align with those reported among other health professionals. Given the relative lack of diversity in the field and rapidly expanding opportunities for genetic counselors to serve in roles and settings in which they are not providing genetic counseling as it is defined, there is risk of some members of the profession feeling excluded from the profession, which in turn could negatively impact the collective identity of the profession, career satisfaction, and patient care. Additional research regarding the PI of genetic counselors is needed.

## AUTHOR CONTRIBUTIONS

The primary author (KS) served as a research assistant to the senior author (LS). She holds a bachelor's degree and post‐baccalaureate certificate in molecular diagnostics. She is planning to pursue a master's degree in genetic counseling. The senior author (LS) is a master's degree‐trained genetic counselor who has not been practicing as a genetic counselor since 2004 and completed her doctorate (EdD) in higher education administration in 2010. She is currently an associate professor who teaches courses in genetics, ethics, and leadership and received the 2023 Jane Engelberg Memorial Fellowship to conduct the study presented. RM is a research genetic counselor affiliated with a master's in genetic counseling training program and completed her doctorate (PhD) in education leadership, policy and human development with a focus on adult, workforce, and continuing professional education. Her dissertation research focused on the professional identity formation of early career genetic counselors. IK is currently a practicing genetic counselor working in an academic setting and holds a doctorate in translational health sciences. All authors participated in all phases of the scoping review and preparation of the manuscript. All authors confirm that they have full access to all the data in the study and take responsibility for the integrity of the data and the accuracy of the data analysis. All of the authors gave final approval of this version to be published and agree to be accountable for all aspects of the work in ensuring that questions related to the accuracy or integrity of any part of the work are appropriately investigated and resolved.

## CONFLICT OF INTEREST STATEMENT

The authors, KS, RM, IK, and LS, declare that they have no conflicts of interest.

## ETHICS STATEMENT

Human Studies and Informed Consent: No human subjects research was carried out by the authors for this scoping review.

Animal Studies: No nonhuman animal studies were carried out by the authors for this scoping review.

## Supporting information


Table S1

## Data Availability

The data that support the findings of this study are available on request from the corresponding author.
