# Supplementary material for: Genetic counselors' professional identity in North America: A scoping review
Source: J Genet Couns. 2024 Jun 11;34(1):e1931. doi: 10.1002/jgc4.1931 (PMC11735250; doi:10.1002/jgc4.1931)
Supplement: Supplementary file 1 — Table S1 [file JGC4-34-0-s001.docx]

Supplementary Table 1. Additional characteristics of full-text articles.

| **Authors** | **Title** | **Purpose of article** | **Type of article** | **Categories** | **Context in which PI is described within the article** |
| --- | --- | --- | --- | --- | --- |
| Abrams & Kessler, 2002 | The Inner World of the Genetic Counselor | Share vignettes that "reflect and examine some of the common themes many GCs face in their work" | Non-empirical research | Reflective practice | Aspects of GC identities are woven into the vignettes. |
| Atzinger et al., 2007 | Characterization of the Practice and Attitudes of Genetic Counselors with Doctoral Degrees | Investigate opinions and attitudes of GCs with doctoral degrees and impact on their careers | Qualitative (e.g., interviews, focus groups) | Professional development | Obtaining a doctoral degree did not significantly change the practice of GCs but the reasons for pursuing further education was congruent among most members. GCs with a doctoral degree spend more time in research and report more respect from colleagues. |
| Bao et al., 2020 | Reflections on diversity, equity, and inclusion in genetic  counseling education | Discuss influence of personal cultural identity in GC profession and need to promote more diversity and equity in the field | Non-empirical research | Diversity, equity, inclusion, & justice | Influence of personal cultural identity when becoming a GC and how the lack of diversity within the professional effects its members/interactions with the community |
| Bartlett & Johnson, 2009 | God and Genes in the Caring Professions: Clinician and Clergy Perceptions of Religion and Genetics | Investigate clinicians’ and clergy’s perceptions of and experiences with religion and genetics in their clinical and pastoral interactions | Qualitative (e.g., interviews, focus groups) | Diversity, equity, inclusion, & justice | Influence of religion and personal beliefs in the GC profession |
| Baty, 2018 | Genetic counseling: Growth of the profession and the professional | Summary of development of genetic counseling as a profession | Non-empirical research | Historical; Identity of the profession | Establishing a graduate program, a national society, and research and scientific publications contribute to the formation of the identity of the profession and ultimately individual professional identity. |
| Bradley, 2012 | Learning to Define Myself | Defining moment where a GC turned her failure and professional identity crisis into a positive experience | Non-empirical research | Single person perspective - Defining Moment article series | Relationship between passing the certification exam and the feeling of being an accepted GC, and its impact on professional identity |
| Cantor et al., 2019 | Genetic counseling students’ experiences with mental illness during training: An exploratory study | Study of experiences of mental illness during training among GC students and recent graduates | Mixed methods | Diversity, equity, inclusion, & justice | Mental illness and self-doubt contribute to the formation of a unique GC identity and the need to live up to the perceived identity of a GC. |
| Carmichael et al., 2021 | Supporting a sense of inclusion and belonging for genetic counseling students who identify as racial or ethnic minorities | Characterize training experiences of GC students who identify as racial or ethnic minorities | Qualitative (e.g., interviews, focus groups) | Diversity, equity, inclusion, & justice | Focuses specifically on experiences that impacted participants’ sense of belonging in the genetic counseling profession and the NSGC conference |
| Channaoui et al., 2020 | Summary report of the 2019 Diversity and Inclusion Task Force of the National Society of Genetic Counselors | Creation of the Diversity and Inclusion Task Force and the recommended charges for creating a more diverse, inclusive, and equitable GC field | Non-empirical research | Diversity, equity, inclusion, & justice | Sense of belongingness and connection to the profession is linked to DEIJ and the inclusivity one feels as part of the profession |
| Curnow, 2012 | Discovering Resilience | Describe a defining moment that fuels purpose of being a GC and deriving value from these experiences | Non-empirical research | Single person perspective - Defining Moment article series; Professional development | Impact of patient with HD's resilience on realization of why am a GC |
| Darr et al., 2023 | Effects of genetic counselor disabilities on their professional experiences: A qualitative investigation of North American counselors' perceptions | Explore academic and professional experiences of GCs who  self-identify as having a disability and their perceptions of whether/how their disability has played a role in their experiences | Qualitative (e.g., interviews, focus groups) | Diversity, equity, inclusion, & justice | Experiences of self-identified GCs with disabilities in relation to sense of belonging within the field |
| Davis et al., 2020 | Genetic counselors with advanced skills: II. A new career  trajectory framework | Professional development is influenced by experiences, colleagues, and self-reflection which influences career changes and advancements. | Qualitative (e.g., interviews, focus groups) | Professional development | The factors that influence professional development also influence professional identity and place/feelings within the profession (belongingness). |
| Disco, 2016 | Lessons from Freelancing, Lighting Design to Genetic Counseling | Individual's story as to why chose genetic counseling as a career path, overlap with previous career, and considerations for GC career progression | Non-empirical research | Single person perspective; Professional development | Defining moments that give reason for pursuing the genetic counseling profession |
| Finucane, 2012 | 2012 National Society of Genetic Counselors Presidential Address: Maintaining Our Professional Identity in an Ever-Expanding Genetics Universe | Presidential address describing core skills and important moments from the GC profession | Non-empirical research | Single person perspective - NSGC Presidential Address; Identity of the profession | Describes some of the different roles GCs have filled but relates each back to the core skills and what it truly means to be a GC |
| Flynn, 2012 | A Couple’s Devastating Journey & My Development as a Genetic Counselor | GCs reflection of working with a family whose husband died of cancer | Non-empirical research | Single person perspective - Defining Moment article series | Defining moment that contributed to GC feeling like a GC and what she values as part of the profession/role |
| Geller et al., 2009 | The Role and Impact of Personal Faith and Religion Among Genetic Service Providers | Describe impact of personal faith and religion on genetic service providers' | Mixed methods | Diversity, equity, inclusion, & justice | Individual's religious beliefs and values may conflict with professional identity and impact their sense of belonging and connectedness within the field, among colleagues, and with patients, resulting in feelings of not belonging. |
| Groepper et al., 2015 | Ethical and Professional Challenges Encountered by Laboratory Genetic Counselors | Examine ethical and professional challenges encountered by GCs working in laboratory setting | Quantitative (e.g., survey, instrument) | Ethics-based | Professional identity issues were among the ethical/professional domain choices in the survey, and then further delineated into categories, one of which is 'professional image.' |
| Hippman & Davis, 2016 | Put Yourself at the Helm: Charting New Territory, Correcting Course, and Weathering the Storm of Career Trajectories | Describe experiences of GCs who made career transitions | Qualitative (e.g., interviews, focus groups) | Professional development | Identifies the high, low, and transition points as a GC changes careers and how that change impacted sense of professional identity and inclusion among other GCs |
| Infante, 2012 | Confusion and Strength | Defining moment that contributed to shaping how this GC practices and her feelings of being a GC | Non-empirical research | Single person perspective - Defining Moment article series | How a defining moment contributes to the identity of a GC.; through impactful experiences, a GC's practice, role, and the feeling of being a GC are formed |
| Knutzen, 2012 | Genetic Counseling Through Hope | Recent GC grad experience with a family whose baby died and concept of hope, as relates to being a GC | Non-empirical research | Single person perspective - Defining Moment article series; Identity of the profession | Questioning and accepting being in GC profession |
| Lakhani, 2012 | It's a Small World: Fusion of Cultures in Genetic Counseling | Defining moment where GC's personal identity is used to connect with patients and shapes her role as a GC in an unfamiliar setting | Non-empirical research | Single person perspective - Defining Moment article series; Professional development | GC's personal identity greatly contributes and shapes her professional identity, shown through a defining moment that shifts her GC practice |
| Liker et al., 2019 | Challenges of infertility genetic counseling: Impact on counselors' personal and professional lives | Personal and professional characteristics of infertility GCs and how their practice impacts their personal lives and professional work | Qualitative (e.g., interviews, focus groups) | Ethics-based; Reflective practice | How experiences shape GC professional and personal decisions, practice, and attitudes |
| Liu et al., 2011 | Diversity and General Student Scholarship Recipient Essays: 2010 National Society of Genetic Counselors Membership Committee | Share student's perspective of the importance of diversity within the GC field and strategies to increase awareness of the profession | Non-empirical research | Single person perspective; Diversity, equity, inclusion, & justice | How the student's see themselves as part of the profession and the impact their background has on who they are as a GC/student |
| Matloff, 2006 | Becoming a Daughter | Defining moment for GC when her mother diagnosed with breast cancer ultimately impacted her practice and perspective | Non-empirical research | Single person perspective; Reflective practice | How a defining moment shapes GC role and sense of self |
| McKanna, 2012 | "Sometimes, it's hard to be a parent" | Explanation of how personal and professional experiences/identities are related and ultimately cannot be separated | Non-empirical research | Single person perspective - Defining Moment article series; Reflective practice | Focus on realization no longer wanted to be a pediatric GC but still seems to identify as a GC - discussion of interaction of professional and personal identities |
| Means et al., 2020 | “I am a Genetic Counselor”: A qualitative exploration of field leaders’ perceptions of the title “genetic counselor” | Interviews of leaders in field regarding the title of GC for the profession | Qualitative (e.g., interviews, focus groups) | Identity of the profession | Focus on the title of the profession but reference to identity of GCs |
| Miranda et al., 2016 | Portrait of the Master Genetic Counselor Clinician: A Qualitative Investigation of Expertise in Genetic Counseling | Description of personal and professional characteristics of 'expert' master GCs | Qualitative (e.g., interviews, focus groups) | Professional development | Common and similar characteristics between professions and expert clinicians which includes a well-defined professional identity, personal values, and professional development for GCs; GCs share similar values and characteristics |
| Oswald, 2012 | Being The Genuine Me | Defining moment for a GC where the experience shaped/changed who she was as a GC | Non-empirical research | Single person perspective - Defining Moment article series; Professional development | The professionaal identity of a GC is shaped and further defined by an impactful moment or case. Specifically, the "calling" this GC felt as someone in this profession and position was highlighted. |
| Paull & Lipinski, 2012 | Taking Advantage of a Unique Opportunity: A Genetic Counseling Fellowship in Lysosomal Storage Disorders | Defining moment that significantly impacted GC's professional development | Non-empirical research | Single person perspective - Defining Moment article series; Professional development | A defining moment that allowed the GC to enhance her professional development while discovering who she was as a GC |
| Peters, 1987 | Feminist Theology and Genetic Counseling: Out of the Closet and Into the Clinic | Describe principles of genetic counseling that align with feminist theory | Non-empirical research | Commentary/Opinion piece/Letter to editors; Diversity, equity, inclusion, & justice; Identity of the profession | Feminist identity and principles as identity of the profession |
| Ramachandra et al., 2019 | An exploration of novice genetic counselors’ transitional  challenges: Commencement is just the beginning | Investigate the transitional challenges GCs experience after leaving graduate school and entering the workforce | Qualitative (e.g., interviews, focus groups) | Professional development | Themes included lack of confidence and imposter syndrome; one participant noted doubts about being an 'actual' GC since in lab role |
| Ramos, 2018 | 2018 National Society of Genetic Counselors Presidential Address | Presidential address speaking to what unites GCs and the motivations they possess for their careers | Non-empirical research | Single person perspective - NSGC Presidential Address; Identity of the profession | Shared values and characteristics of GCs and members of NSGC |
| Riordan, 2021 | 2021 National Society of Genetic Counselors Presidential  Address: Seeking opportunities for growth and leading each  other | NSGC presidential address | Non-empirical research | Single person perspective - NSGC Presidential Address; Professional development; Identity of the profession | Described elements of her identity that influenced her work and how her work as a GC has shaped her; she also references the identity of the profession in relation to efforts of NSGC |
| Runyon et al., 2010 | What Do Genetic Counselors Learn on the Job? A Qualitative Assessment of Professional development Outcomes | An explanation of professional development within the GC profession and advice for students | Quantitative (e.g., survey, instrument) | Professional development | Speaks to what genetic counselors learn about themselves while working as a GC |
| Schoonveld et al., 2007 | What Is It Like To Be in the Minority? Ethnic and Gender Diversity in the Genetic Counseling Profession | Discuss impact of being an underrepresented minority in the GC profession while highlighting factors that contribute to barriers or success of entering the field | Qualitative (e.g., interviews, focus groups) | Diversity, equity, inclusion, & justice | The relationship between cultural identity, gender, and socio-economic status and feelings of belongingness in the profession. |
| Strohmeyer et al., 2023 | No longer “non-traditional”: Genetic counselors' perceptions towards laboratory and industry roles | Assess characteristics of laboratory and industry roles and perceptions among GCs serving in these roles, particularly as it relates to stigma and biases experienced being in a "non-traditional" role | Quantitative (e.g., survey, instrument) | Professional development | GCs in "non-traditional" roles were experiencing professional identity issues, stigmatization, and overall not feeling like a GC because of their job position. |
| Sturm, 2019 | 2019 National Society of Genetic Counselors Presidential  Address | Presidential address motivating the unity of the profession | Non-empirical research | Single person perspective - NSGC Presidential Address; Identity of the profession | Characteristics of GCs within the profession like "pioneers" and "leaders" |
| Uhlmann, 2012 | National Society of Genetic Counselors Natalie Weissburger Paul Lifetime Achievement Award Address: The Power of Connecting | NSGC awardee address | Non-empirical research | Single person perspective - NSGC Awardee Address; Identity of the profession | Outline of contributions to GC field and NSGC, as well as reasons for becoming a GC |
| Wells et al., 2016 | Development, Experience, and Expression of Meaning in Genetic Counselors’ Lives: an Exploratory Analysis | Investigate how meaningful moments contribute to GC's professional development and how "meaning making" is developed/shaped and the outcomes | Qualitative (e.g., interviews, focus groups) | Professional development; Reflective practice | Influence of meaning making on GCs and the outcomes of those situations both negative (burnout, compassion fatigue, etc.) and positive (professional development) |
| Zahm et al., 2016 | From Novice to Seasoned Practitioner: a Qualitative Investigation of Genetic Counselor Professional development | Investigate professional development of GCs and its relationship to their role, field, and identity | Qualitative (e.g., interviews, focus groups) | Professional development; Reflective practice | Impactful and defining elements of the GC profession contribute to the sense of being a GC and ultimately contribute to their PI and development. |
| Zetzsche et al., 2014 | Looking Back and Moving Forward: An Historical Perspective from Laboratory Genetic Counselors | Describe evolving role of GCs in laboratory setting and explore the opinions and feelings of laboratory GCs and how they see themselves as part of the profession. | Qualitative (e.g., interviews, focus groups) | Professional development | How a non-clinical role impacts PI and sense of belonging to the field along with recognition from others |
